# Beyond crystal balls: crosscutting solutions in global health to prepare for an unpredictable future

**DOI:** 10.1186/s12889-015-2285-1

**Published:** 2015-09-24

**Authors:** Wladimir Jimenez Alonso, Benjamin Joseph James McCormick, Mark A. Miller, Cynthia Schuck-Paim, Ghassem R. Asrar

**Affiliations:** Fogarty International Center, National Institutes of Health, Bethesda, Maryland 20892 USA; Origem Scientifica, São Paulo, São Paulo Brazil; Joint Global Change Research Institute, University of Maryland, College Park, MD 20740 USA

**Keywords:** Public health, Prediction, Models, Resilience, Health risks

## Abstract

**Background:**

Efforts in global heath need to deal not only with current challenges, but also to anticipate new scenarios, which sometimes unfold at lightning speed. Predictive modeling is frequently used to assist planning, but outcomes depend heavily on a subset of critical assumptions, which are mostly hampered by our limited knowledge about the many factors, mechanisms and relationships that determine the dynamics of disease systems, by a lack of data to parameterize and validate models, and by uncertainties about future scenarios.

**Discussion:**

We propose a shift from a focus on the prediction of individual disease patterns to the identification and mitigation of broader fragilities in public health systems. Modeling capabilities should be used to perform “stress tests” on how interrelated fragilities respond when faced with a range of possible or plausible threats of different nature and intensity. This system should be able to reveal crosscutting solutions with the potential to address not only one threat, but multiple areas of vulnerability to future health risks.

**Summary:**

Actionable knowledge not based on a narrow subset of threats and conditions can better guide policy, build societal resilience and ensure effective prevention in an uncertain world.

## Background

In the last 40 years the human population has almost doubled [1]. Massive concentrations of people in mega-cities, rapid changes of land use and competition for natural resources, fertile land and energy sources are all growing challenges [2] that already consign a disturbing proportion of humanity to hardship and poverty. Almost half the world’s population live on less than $2.50 a day [3] and every day around 21,000 children die [4] due to preventable diseases [5]. On top of all this, environmental, socioeconomic and geopolitical conditions we take for granted can change, and may exacerbate the already daunting challenge of a healthy and productive life for all.

There have been substantial efforts to forecast these changes and predict their consequences to guide preparedness and preventive efforts in public and global health systems. Prediction of the likelihood of disease outbreaks, the emergence of new diseases, geographic areas at risk, disease spread, and the timing and severity of disease events has indeed achieved a prominent role in epidemiological and global health research.

However, reliance on predictive models can be dangerously misleading given the many sources of uncertainty associated with predictive modeling exercises. The reduction of disease systems to key components for the purpose of an abstracted mathematical model may reflect only partial understanding that may not include pertinent factors. Model parameters may behave with wide margins of poorly characterized variability. Important features of a system may be also missing, Hypothetical future scenarios may be specified inappropriately and novel conditions or non-linear dynamics may render the past an unreliable guide to the future. Also, influential but rare conditions are often missing from the input data used in modeling efforts. Additionally, models can only rarely be tested, as they are simplified representations of complex and interdependent relations among factors that may vary in time and space. These limitations have been articulated strongly for environment, energy and economics [6] fields in which long term predictions are very important, but they are equally applicable to the health sector.

Here we argue for a shift in epidemiology and global health research that orientates the formulation of research questions and integrates interdisciplinary expertise to address complex health issues and guide the development of practical solutions. This interdisciplinary framework is less reliant on the uncertain exercise of predicting specific risks associated with novel scenarios or rare events and conditions. Instead, it focuses on the identification of interventions most likely to mitigate fragilities and build resilience among populations and public health systems, increasing their ability to adapt to changing circumstances [7, 8] and emerging threats.

## Discussion

### Predictive modeling in global health and epidemiology

Models are simplified abstractions designed, with particular questions, to help understand key components of a system or to evaluate scenarios in which those processes or components change. Because they condense current knowledge into explicit and tractable forms, they have been productively used to tease apart relationships and risk factors in many disease systems [9–11]. They can also qualify some forms of uncertainty in current knowledge, thereby assisting decisions to manage risks under resource constraints [12]. With advances in computing power and the adoption of massive shared data [13, 14], as adopted for example by the Global Burden of Disease initiative [15], inputs to models are improving, as are existing modeling approaches with the use of techniques borrowed from different disciplines. For instance, machine learning methods can be used to extract information, simulation modeling enables better use of observed data and the possibility to run hundreds of *in silico* experiments [16] and model averaging approaches [17] can help to describe and account for variability both from data and from structural uncertainty in existing models. These methods, however, still depend on the quality of biological assumptions and observations [18].

Modeling efforts are, however, constrained by our current understanding of the processes investigated, hence by the suitability of assumptions made. That is often the case with phenomena involving many variables that behave in uncertain ways (e.g., the spatio-temporal dispersion of a newly emerging disease). First, key factors and covariates may be unknown or missing [19]; input data to train models may be also lacking or limited; and the suitability of surrogate variables may be uncertain. Sampling variability and measurement errors further add to the uncertainty. Because epidemiological models are developed using historical observations that often exclude rare events, our ability to predict new conditions of relevance (crises, outbreaks, environmental changes and other disasters) or the cascading consequences of extreme events is limited. Incomplete knowledge of system dynamics and mediating mechanisms may often lead to exclusion of important variables, interactions and improper choices during model design. The uncertainty derived from the multiple choices involved in data processing and model construction (e.g., type of model, dynamic aspects, spatio-temporal scale, discrete or continuous nature of variables, stochasticity, level of analysis and the various statistical choices) is also often unaccounted, despite their direct effects on model estimates [20]. Additional sources of uncertainty come into play when model outcomes are extrapolated into the future, including poor specification of hypothetical future scenarios and the uncertainty or suitability of assuming that the past can mirror the future reliably. Finally, verification and validation to determine the credibility of outcomes is only rarely conducted (or impossible given the infrequency and unique challenge of each health outbreak), instead relying on post-hoc rationalization, yielding a false impression of predictive credibility [6, 21, 22].

Consider for example the shortcomings associated with the use of predictive modeling approaches in two of the most widely studied disease systems in global health: influenza and malaria. Although influenza is one of the most studied infectious diseases of our time, we still lack an explanation for the basic mechanisms driving influenza disease burden during regular (seasonal) epidemics in different climates and regions [23]. Accordingly, accurate actionable predictions are both difficult to generate (e.g. [16, 24]) and rarely tested [25, 26]. Although models of influenza dynamics are common, predictions are often limited in their time and geographic horizons as well as their ability to distinguish the burden of illness associated with other respiratory pathogens and disease drivers [27–29]. The challenge is made harder when one moves beyond predicting regular events (e.g. seasonal dynamics) to predicting the features of rare events, such as the magnitude, duration, location or time when a pandemic might emerge [30]. Although predictive models of influenza pandemics abound, important aspects such as transmission dynamics and behavioral factors are often missing (reviewed in [31]). Additionally, given the rare nature of these events, modeling outcomes are rarely validated [31] and parameter choices are often based on past studies, not on independent data [31]. Importantly, the ability to specify the dynamics of putative future pandemics appropriately is further hindered when we consider that emergence of new viral strains is inherently unpredictable [32, 33].

A second example is the forecast of disease distribution as a function of climate, an approach widely adopted in the development of risk maps of malaria [34, 35] and other diseases [36–38]. These models attempt to identify past climatic conditions that best discriminate between the observed presence of a disease and theoretical areas of absence [39]. The model then projects this statistical association between climate and the current or historic disease distribution into the future. Nevertheless, the ecological space occupied by a disease at present is heavily influenced by historical factors other than climate [40, 41], such as efforts to actively eliminate the disease. Therefore existing spatial distributions (the “realized niche”) are, in fact, a subset of the ecological space that the species could occupy (the “fundamental niche”) [39]. Areas assumed to be climatically unsuitable for malaria (absence points) may be in fact suitable, impairing the predictive ability of this exercise. This exemplifies how uncertainties about even a single factor (of the many possible) can affect model predictions and the policies that derive from them. Not surprisingly, forecasts on the effect of climate change on malaria transmission have been shown to be spatially heterogeneous and largely inconsistent [42].

A third and more recent example illustrates how data from an unusual outbreak (in contrast to the well described epidemiology of malaria and influenza) were used to build short-term forecasts. The 2014–2015 Ebola outbreak in West Africa rapidly led to several models forecasting the size of the epidemic and evaluating alternative interventions [43–50]. The majority of these models were variations on an existing framework [51], but whether they were deterministic models or attempted to account for random stochastic chance, these models all over-estimated the epidemic [52, 53]. This was additionally noteworthy because the assessment of model accuracy was conducted not by the model developers, but by journalists. In terms of what we could learn from the models, the interventions evaluated (e.g. hygiene practices, funeral arrangements and availability of hospital beds) were not novel and their lack of implementation was more likely a symptom of a weak health infrastructure and execution than some failure related to this individual outbreak [54, 55]. Indeed, a general improvement to health access and provision would not only have had a dramatic positive impact on the Ebola outbreak, but also addressed other health fragilities [56, 57]. Though difficult to implement (e.g. [58]), investment in wider fragilities would have even wider reaching benefits.

In the following section we propose an alternative framework for health research and policy formulation.

#### An alternative framework to guide health research and policy

The limitations of predictive endeavors are not an impediment to directing efforts to lower the impacts of current and putative threats. We argue that we need to shift the focus from the prediction of specific health breakdowns, reliant on accurate and detailed knowledge of risk factors, to the identification of broader points of fragility within communities, regions and societies. A similar debate has been on-going in the climate change literature as to whether prevention strategies ought to be based on the predictions of a small number of hypothetical future scenarios or if the response to present and past conditions should be adaptive to uncertain future change [12].

**Fig. 1:**
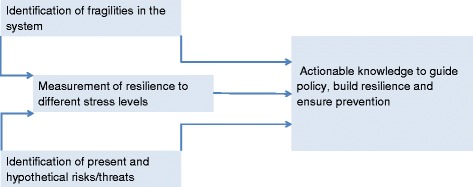
Suggested framework to analyze the fragility of public health systems given uncertainty about potential stressor with the goal of enabling more actionable and effective interventions

We suggest identifying points at which a society is fragile to changes in broad causes of stress or inter-related family of stresses. We prefer to be deliberately inclusive in characterizing stressors that are capable of impacting negatively (directly or indirectly) the provision of public health. For example, one such stressor might be climate, another may be military conflict and another could be fluctuating economics. The practical outcome is a more immediately manageable picture for public health decision makers. Importantly, we do not suggest the common practice of devolving identification of actionable responses from model construction. Instead, the approach proposed is based on three layers shown in left hand side of the diagram of Fig. 1. Because focusing on the likelihood of a particular event might ignore the fragility of public health to more general classes of perturbation, preparedness plans focused on specific risks might do little to reduce the overall fragility of a health system to multiple stressors. Therefore, efforts should be biased toward identification of points of fragility associated with maximal cascading challenges for public health, and on the evaluation of cross-cutting and flexible solutions for an uncertain future. Greater societal resilience to known and unpredictable disease risks can be similarly achieved by the active identification and mitigation of fragilities to a wide range of stressors. A broader framework for policy and research should also enable trans-disciplinary collaboration, reducing public spending on multiple fronts by preventing of simultaneous efforts as much as possible.

#### Identification of fragilities within public health systems

A vast number of pathogens can affect humans, and some operational classification is necessary to identify generalized fragilities. This is a non-trivial challenge [59]. However, a convenient start can be the International Statistical Classification of Diseases and Related Health Problems [60], which groups disease causes hierarchically based on shared anatomical and physiological features. Knowledge of grouped threats can directly inform research and policy: for example, a community that does not generally adopt safe sexual practices is fragile to STDs. We require neither knowledge that pathogens responsible for STDs are circulating nor indeed data of the precise disease burden in current circulation to make the case that there is a potential (future) risk. Of course, the more comprehensive the available information is, the better the assessment of differential outcomes in response to variable levels of exposure (see below). Efforts such as the Global Burden of Disease [15] to compile and make available massive and robust standardized data sets offer the opportunity to capitalize on significant efforts to gather detailed information to establish health status and explore common risk groups.

**Fig. 2:**
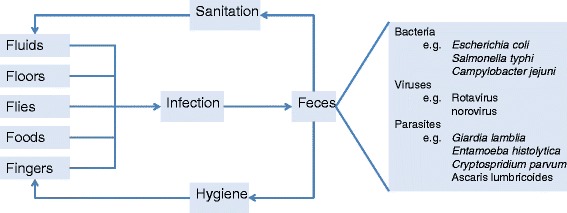
The “F-diagram” clustering routes of transmission for diarrheal pathogens [72]. WASH (water, sanitation and hygiene) programs attempt to tackle the common routes of infection rather than separately address individual pathogens

An excellent example of a health fragility is illustrated by the Mills-Reincke phenomenon [61]. In the 1900s, improvements to municipal water supplies successfully reduced the burden of mortality from typhoid disease – the motivating target for the action. However, all-cause mortality was also reduced (saving between 1.5 to 16 times the number of lives from combined ‘all cause’ compared to the number saved from typhoid alone), as was substantial indirect morbidity with more recent estimates that around 4 % of all deaths might be directly attributable to water/sanitation [62]. At the time the routes of infection and breadth of individual pathogens were unknown, but the same logic is widely applied in WASH (water, sanitation and hygiene) programs that attempt to block routes of infection (Fig. 2) rather than attempt to focus on individual pathogens – of which there are many [63]. WASH programs not only tackle fragilities to multiple enteric infections, but they focus on proximate stressors (e.g. water quality at the point of use, or household hygiene) rather than ultimate stressors that might, for example damage the supply of clean water.

#### Identification of present and potential proximate stressors

The probability that pathogens can successfully establish an infection and the pathological consequences thereof are frequently so inter-dependent with environmental conditions that we might choose to operationally waive individual pathogens as “stressors”, and instead concentrate on those conditions that permit them to harm public health (important exceptions to this assumption include the introduction of highly transmissible and deadly pathogens; in such cases, it is more appropriate to consider pathogens themselves as stressors). The identification and systematization of exposure conditions and potential threats is fundamental to characterize fragilities within a system. This task requires multidisciplinary calls for linking as many as physical, physiological, social, epidemiological and geopolitical processes as possible.

To simplify stressors into operational units, it is important to discriminate them as “proximate” or “ultimate”, namely those with direct or indirect consequences for public health, respectively. We suggest focusing on the former, since their immediate effect on health makes them more readily identifiable. For example, *per capita* water shortages represent one such proximate stressor. It may have different causes, such as the contamination of reservoirs, increase in price, a weather related event, local or global climatic changes or a terrorist act, all of which are ultimate stressors (i.e. these stressors are one step removed from public health; their effect is felt via the proximate stressor – water shortage). Since different events can lead to water shortage, instead of trying to predict which could trigger a current or future shortage event, it would be more effective to examine whether and how much proximate public health stressors (in this case, water shortage) can impact populations, and prepare for it accordingly.

In some cases the relationship between stressors and health fragility is relatively simple and well described. Air quality is, for example, one of the main environmental factors of concern for acute lower respiratory infections (ALRI) [64]. Thus, information about air quality – the proximate stressor – that is routinely collected for other purposes, provides a reasonably accurate approximation of where the population is likely to be susceptible to ALRI without necessarily requiring detailed knowledge of ALRI itself (surveillance of which may not be routine or might only be for symptoms, by which time people are already infected). In other cases, the association between risk factors and health outcomes is more opaque. For instance, an increase in winter temperatures may permit bacterial growth over a longer period of the year, increasing the risk of enteric disease. Simultaneously, warmer winters may reduce survival of respiratory viruses or temperature-induced injury to host defenses [23], thereby reducing the stress imposed on the respiratory system.

Focusing on proximate stressors avoids the uncertainty about which event (ultimate stressor) will impact public health since we concentrate on immediate chains of events. Careful identification of proximate stressors can reduce the many different sources of public health fragility into a lower number of factors likely to be more readily evidenced than specific public health challenges.

#### Assessment of system resilience to different stress levels

Where stressors can be broken into more refined qualitative (better yet, quantitative) levels, an analytical refinement is to estimate the degree of resilience or fragility of populations to varying levels of exposure. For example, maps that identify ‘hotspots’ of stress based on the intersection of layers of information for different sources of societal fragility together with environmental stressors can provide a powerful means for policy makers to interpret either realized or plausible scenarios characterized by gradual or unexpected disruption to services in response to changes in one or more layers of stress (with due consideration to demography of affected sectors of society). A recent example is the mapping of hotspots of emergent diseases to highlight surveillance needs based on the overlap between areas of high human population density, the presence of animal reservoirs and specific climatic conditions [65].

An exciting and particularly useful exercise is the identification of mechanistic links between stressors (Fig. 1) that can inform the classification (or gradation) of potential stressors and identify ways that changes at one layer may cascade across others (e.g., the economic consequences of human disease on conflict and health [61, 66]). Cost-benefit analysis of alternative strategies that use these cross-linkages of multiple fragilities may suggest solutions that would not be apparent if fragilities are examined in isolation. An example is the introduction of remote consultation by telephone or email for health care of low-risk patients [67, 68]. Through an improved ability to use remote communication for medical care, such a system not only reduces fragility to infectious diseases transmitted in public settings (e.g. influenza in hospital waiting rooms), it would also enable populations to gain ‘anti-fragility’ [21] to a wider range of stressors (e.g., it could reduce burden in other contexts, such as the aftermaths of earthquakes, civil unrest and other disruptions to infrastructure).

## Conclusions

Predictive modeling of complex systems has inherent weaknesses that stem from extrapolating current circumstances into the future, ignoring knowledge limitations about key components of the system and their interactions, their dynamics and driving factors and from difficulties to test and falsify predictions as their trial is set ahead in the future (exceptions include the fields of meteorology, elections and sports, where models are often based on a short enough time frame and regular occurrence to allow validation).

We propose the identification of public health fragilities – which may result from current stressors, predicted trends or a range of potential shocks – as an important target for public health models. Policies responding to future forecasts concentrating on these health system fragilities would be more robust to uncertainty than case-by-case models. The rhetoric of “health in all policies” in cross-organizational policy organization is not always translated into action, since preventative or promotion of health gets diluted amongst several specific initiatives or becomes an independent silo [69]. Our proposed approach resembles more the adoption of “all policies in health” by the modeling and research community in an effort to move away from predictive models of individual and specific health risks and toward models that encompass health fragilities within broader societal systems [7, 70].

By extending this framework across different categories of risk, strategies can be devised that account for different sources of exposure, mitigating more effectively the cascading consequences of unforeseen extreme events. Instead of predicting specific conditions or risks in the future, the approach proposed here builds on recent efforts concentrated on climate [71] to deal with a range of plausible scenarios, testing the resilience of a system in face of an unpredictable, though predictably uncertain, future.
